# Agreement and reliability of dairy cow udder morphometrics between traditional measurements and measurements from a 3-dimensional scanner

**DOI:** 10.3168/jdsc.2025-0822

**Published:** 2025-10-25

**Authors:** J.M. Strickland, C.I. Robison, P.H.E. Trindade, P.L. Ruegg

**Affiliations:** 1 Department of Large Animal Clinical Sciences, College of Veterinary Medicine, Michigan State University, East Lansing, MI 48824; 2 Department of Animal Sciences, Michigan State University, East Lansing, MI 48824

## Abstract

The morphology of the dairy cow mammary gland can indicate risk of disease and can change in response to various conditions. Collecting traditional measurements with a tape measure is time-consuming and can be subject to observer error. The objective of this cross-sectional study was to assess the agreement and reliability between traditionally collected measurements of the hind udder with measurements collected using a 3-dimensional (3D) scanner. We hypothesized there would be good reliability and agreement between methods. Adult cows in tiestalls at the Michigan State University dairy farm were used (n = 40). On 3 consecutive days, trained observers (n = 3) used a soft measuring tape to perform 3 measurements of the udder: a single vertical measurement from the rear udder attachment to the base of the quarter, and separate horizontal measurements of each quarter (left and right) from the medial cleft to the hind leg juncture. Marks were placed on the udders to guide traditionally collected measurements. A separate observer collected 3D scans (Structure Sensor, Occipital) and measured the same areas using 3-matic software (n = 120; Materialise, Belgium). Cows with mastitis (n = 3) and one incomplete scan were removed from the data, resulting in 37 cows with 999 and 333 traditional and 3D measurements, respectively. Intraclass correlation coefficients (ICC) among observers and between traditionally determined measurements and 3D measured were analyzed. Bland–Altman analysis was used to assess bias between traditional and 3D measurements. Linear regression was used to assess proportional bias between methods. The interobserver reliability was good to excellent for traditional measurements (ICC ≥0.88). Reliability between traditional and 3D measurements was good to excellent for all observers (ICC ≥0.87). Bland–Altman assessment found minimal bias between traditional and 3D measurements, except for 1 observer measurement of the right quarter. As compared with measurements from the 3D scanner, observers A and C consistently underestimated vertical measurements and overestimated the quarter measurements. Of results, 93% to 96% were within the upper and lower bounds of agreement. Observer C had positive proportional bias for vertical and right quarter measurements, indicating that as udder size increased, traditional measurements were overestimated as compared with 3D. These results indicate that the 3D scanner has a good to excellent reliability and adequate agreement with minimal bias in relation with the traditional measurements and may be used to collect hind udder measurements in healthy cows. These methods could be used to improve detection and assessment of conditions such as mastitis, though more research and validation studies are needed.

## Highlights

• The measurements among 3 trained observers had good to excellent reliability.
• There was good to excellent reliability and agreement between tape measure and 3D measurements.
• One observer overestimated udder measurements as they increased in size.

## Graphical Abstract

**Figure:**
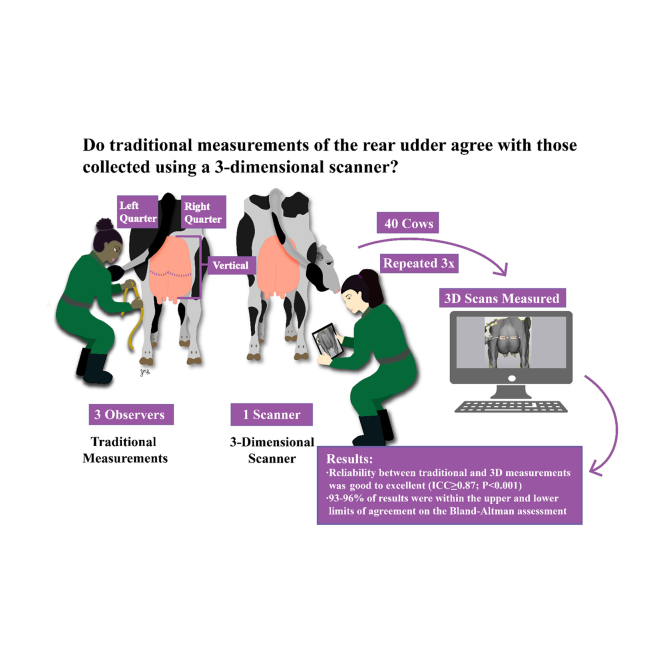
**Summary:** The reliability and agreement of traditionally collected measurements with measurements collected using a 3-dimensional (3D) scanner were assessed on 3 locations on the rear udder of 40 Holstein dairy cows. Vertical measurements were collected from the rear udder attachment to the lowest point of the quarter (solid line). The left and right quarters were each measured from the rear udder cleft to the hind leg juncture along the curve of the udder (dashed lines). Trained observers (n = 3) repeated all udder measurements and collected 3D scans on 3 consecutive days. The scans were measured using the same anatomical locations. Results showed good to excellent reliability and minimal bias between manual and 3D measurements. These results indicate that handheld 3D scanners can be used to collect reliable measurements of the rear udder. Drawn by J. Strickland in Adobe Illustrator I (version 30.0).

The dairy cow mammary gland is composed of dynamic tissue whose morphology can be altered by disease, stage of lactation, milking equipment, and involution after dry-off. The overall morphology of the udder and teats have interested researchers for decades and some characteristics have been shown to contribute to increased risk of mastitis. Miles et al. (2019) reported that loose fore udder attachment and lower rear udder height were associated with increased odds of clinical mastitis as compared with strong fore udder attachment and intermediate rear udder height (Miles et al., 2019). The odds of developing mastitis have been shown to increase as the diameter of the teat apex increases (Guarín and Ruegg, 2016). Flat right hind teat ends and flat right teat ends had greater odds of increased SCC and mastitis, respectively, compared with teats ends that were rounded (Miles et al., 2019). Changes in udder and teat morphology can indicate various mammary gland pathologies that may affect udder health. Increased distention between rear teats and increased teat diameter after dry-off as compared with during lactation indicate increased udder engorgement, which can be lessened by using practices such as reduced milking frequency (Larsen et al., 2021). The effects of milking frequency on udder volume have even been measured by creating foam casts of the udder (Stelwagen and Knight, 1997).

Collection of udder morphology data can provide valuable insight regarding mammary gland health and function, but traditional measurements of the udder are time-consuming and may be subject to observer error (Sapp et al., 2003). Collection of morphometric data has become quicker and easier with the use of 3-dimensional (**3D**) imaging technologies. In the human medical field, 3D scanner use has increased greatly over the last couple of decades and has a wide variety of applications such as collecting anthropomorphic measurements to creating custom implants and prosthetics (Haleem and Javaid, 2019). In dairy production, BCS collected from Holstein cows using a 3D scanner were 3 times more repeatable as compared with traditionally obtained scores (Fischer et al., 2015). Multiple mounted 3D scanners can be used to scan the entire cow to automatically collect body measurements (such as heart girth and withers height) that have otherwise required traditional measurements (Le Cozler et al., 2019). Simplified collection of such measurements could be used to assess growth in lactating animals throughout lactation and to estimate BW (Le Cozler et al., 2019). The use of 3D scanners has been reported to score lameness and to identify specific hoof lesions with less success (Schlageter-Tello et al., 2018; Kang et al., 2021).

Mammary gland dairy type traits collected using 3D cameras have been compared with those collected by an official Brazilian Holstein Association classifier and had correlations between 0.94 for udder depth to 0.5 for rear udder attachment (Martins et al., 2020). Milk yield prediction was assessed when udder volume was estimated using a 3D camera positioned so that cows walked over it before entering the milking parlor (Shorten, 2021). Although several studies have assessed potential uses of 3D cameras in dairy production, no studies have evaluated reliability and agreement for measurements of the udder. Though reliability and agreement assessments are fundamental concepts in evaluating measurement error, they are frequently misunderstood (Kottner et al., 2011). Reliability is the ratio of variability between measurements of the same subjects by different observers to the total variability of all the measurements in the sample (Kottner et al., 2011). Reliability scores how well the subjects being measured can be distinguished from each other by their measurements (Hernaez, 2015). Agreement is how much 2 different methods of measurement differ and the degree of measurement error is the variable of interest (Kottner et al., 2011).

To understand potential applications of using 3D scanners to record udder morphometric data, agreement and reliability must first be established relative to traditional measurements. The objective of this study was to assess the reliability of measurements collected among observers and to determine the level of agreement between traditional measurements of the hind quarters with those obtained from a 3D scanner. We hypothesized that there would be good reliability and agreement between traditional and 3D measurement methods.

This cross-sectional study was conducted at the Michigan State University (**MSU**) Dairy Cattle Teaching and Research Center from December 1 to 7, 2024. A convenience sample of 43 adult Holstein cows that were housed in tiestalls was available for this study. The exclusion criteria included presence of clinical disease 1 wk before the beginning of the study or lacking 4 functional quarters, resulting in 3 cows that were excluded due to diagnosis of clinical mastitis just before the study. Cows with mastitis were excluded to reduce potential error that may occur due to inflammation that could lead the quarter size to vary between measurement collections. In addition, the MSU dairy farm is a National Dairy Quality award winner and achieving an adequate sample size would have been difficult. The 40 enrolled cows were housed in tiestalls on mattresses with wood shavings and were milked 3 times daily. Before performing measurements, rear udders were marked with permanent marker at the rear udder attachment and at the center of each mammary gland quarter halfway between the rear udder attachment and the base of the lowest quarter by the same individual measuring the 3D scans. The observers were trained for measurement collection using the marks as guidance. These marks remained for all traditionally collected measurements and helped to ensure that the same landmarks were used for measurements on the 3D images. Udders were measured vertically from the rear udder attachment to the lowest point of the udder in a straight line. At the halfway point, the curve of each quarter was measured horizontally from the medial suspensory ligament to the hind leg juncture. Traditionally collected measurements were collected using a soft measuring tape in centimeters to the first decimal point. Each observer (n = 3) measured each cow once daily for 3 consecutive days. Data were collected at the same time each day starting 3 h before the final milking of the day. To reduce variability in udder size based on time until milking, measurements were collected on 21 cows the first 3 d of the week and then the remaining 19 cows the final 3 d of the week. Cow posture was adjusted so that all cows were standing squarely with all feet on the stall mattresses. Observers collected measurements independently. During measurement, 1 observer performed 3D scans of the rear udders using a Structure Sensor (Mark II) scanner (Occipital, Boulder, CO) attached to a fourth generation iPad Air (iPadOS 18.1.1, Apple, Cupertino, CA). The 3D scans were exported using the Structure Capture application (version 2.1.0, XRPro LLC, Saratov, Russia). Before scanning, the sensor scanner was calibrated using the Structure Sensor Calibrator application (version 4.5.2, XRPro LLC, Saratov, Russia). Scans were performed from approximately 1 m behind each cow with the scanner held at the level of the udder and the tail held to the side. The scan was completed by slowly rotating the scanner horizontally to capture the sides of the udder that extend beyond the posterior end of the hind legs as well as vertically to ensure the udder was scanned completely including the rear teats and the rear udder attachment.

The 3D object files were imported into 3-matic software (version 19.0, Materialise, Leuven, Belgium) using a scale factor of 1,000 to scale meters to millimeters. The vertical measurement was collected using the distance tool with only the Y-direction selected. The vertical measurement began at the level of the rear udder attachment and ended at the most ventral part of the lowest quarter. The measurement was taken over the medial udder ligament. The length tool was used to collect the quarter horizontal measures with the distance over surface, true shortest path, and world coordinate options selected. Each quarter was measured separately. To ensure that this point corresponded with the marks on the udder, each measurement began at the medial udder ligament and extended horizontally to the juncture of the medial hind leg at the 50% point of the vertical measurement.

Statistical analysis was done using RStudio (version 4.4.1, RStudio Inc., Boston, MA). The packages and functions used were described as ‘package::function’ in accordance with the R computing language. To evaluate interobserver reliability, intraclass correlation coefficient (**ICC**) between observers and between traditional and 3D measurements were analyzed using the irr::icc with a 2-way model with type consistency. The blandr::blandr.statistics and blandr::blandr.draw were used to assess bias between traditional measurements from each observer and the 3D scanner. Linear regression models were created with stats::lm to assess for proportional bias between traditional and 3D measurements for each observer. The dependent variable was difference between traditional and 3D measurements, and the independent variable was the mean of the traditional and 3D measurements. For each linear model, the distribution of residuals was assessed with the Shapiro–Wilk test (stat::Shapiro.test), histogram (stat::hist), and quantile-quantile plots (stat::qqnorm) to assess whether the assumptions of normality had been violated (data not shown). Goodness of fit was assessed with the Cramer-Von Mises test (goftest::cvm.test; data not shown). The levels of reliability were determined using the guidelines outlined by Koo and Li (2016) so that ICC values less than 0.5 indicate poor reliability, values between 0.5 and 0.75 indicate moderate reliability, values between 0.75 and 0.9 indicate good reliability, and values greater than 0.9 indicate excellent reliability.

A few considerations were used in calculating sample size. According to Koo and Li (2016) 2016, reliability studies should have at least 30 heterogeneous subjects, 3 independent observers, and conduct each measurement 3 times. To verify the sample size we used data from a previous study that collected rear udder measurements using both a tape measure and a 3D scanner (unpublished). Using SD of 6.3, power of 80%, α of 5%, and noninferiority limit of 5, we calculated that we needed 40 subjects. Additionally, the International Organization for Standardization recommends a sample size of 40 to determine if a difference exists between traditional forms of measurements such as a tape measure and a 3D scanner (ISO:20685-1; ISO, 2018).

Of the 43 cows available for this study, cows were excluded for having clinical mastitis (n = 2) or for having 3 functional quarters (n = 1). The 40 enrolled cows ranged in parity from 1 to 7 with a mean of 2.8 ± 0.30 and median of 2 ± 0.30. Cows ranged from 77 to 325 DIM, a mean of 156.6 DIM ± 7.63, and a median of 147.5 ± 7.63 DIM. Average daily milk production ranged from 32.7 to 67.3 kg, a mean of 101.6 kg ± 1.45 kg, and a median of 98.5 ± 1.45 kg. The SCC ranged from 13,000 to 187,000 cells/mL, a mean of 30,400 ± 6,396 cells/mL, and a median of 13,000 ± 6,396 cells/mL. During the study, 3 additional cows were diagnosed with clinical mastitis and were removed from analysis. One vertical measurement was deleted before analysis because the image did not include the point of the rear udder attachment, and thus the vertical measurement could not be measured accurately.

The interobserver reliability for the vertical measurements was 0.88 (95% CI: 0.84, 0.92; *P* < 0.001) between observers A and B, 0.9 (95% CI: 0.85, 0.93; *P* < 0.001) between observers A and C, and 0.88 (95% CI: 0.83, 0.92; *P* < 0.001) between observers B and C. The interobserver reliabilities for the right quarter measurements were 0.95 (95% CI: 0.92, 0.96, *P* < 0.001) between observers A and B, 0.93 (95% CI: 0.9, 0.95, *P* < 0.001) between observers A and C, and 0.93 (95% CI: 0.9, 0.96, *P* < 0.001) between observers B and C. The interobserver reliabilities for the left quarter measurements were 0.93 (95% CI: 0.9, 0.95, *P* < 0.001) between observers A and B, 0.91 (95% CI: 0.9, 0.94, *P* < 0.001) between observers A and C, and 0.9 (95% CI: 0.86, 0.93, *P* < 0.001) between observers B and C.

The interobserver reliability between each of the 3 observers and the 3D scanner for the 3 different udder measurements ranged from good to excellent (Table 1). Whereas 2 of the 3 observers had excellent reliability with the 3D scanner for the vertical and right quarter measurements, all 3 observers only had only good reliability between their measurements and the 3D scanner for the left quarter measurements. All 3 observers in this study were right-hand dominant, which may explain why the ICC values of the left but not the right quarter were all below 0.9 when compared with the 3D scanner. Handedness of observers can affect measurements of live animals and even on images of animals, so it should be considered in future studies (Helm and Albrecht, 2000; Van Nuffel et al., 2007). The bias between traditional and 3D measurements was minimal (<1) with the exception of the right quarter measurement between observer A and the 3D scanner (Table 1). Additionally, the majority of data points from the Bland–Altman assessment were between the upper and lower limit of agreement (Figure 1). There were 2 cows whose measurements fell outside of the limit of agreement 5 or more times and that was likely because they moved often, making both methods of measurements more difficult. The data points for these 2 cows occurred above and below the limits of agreement with no discernable pattern. There was a proportional bias for observer C with the vertical and right quarter measurements (*P* = 0.03). For these 2 measurements, as the size of the udder increased, observer C's measurements were overestimated compared with the 3D scanner as illustrated by the upward pattern of the data points and linear regression plot (Figure 1).

**Table 1:** Interobserver reliability and Bland–Altman agreement results between traditional measurements and the 3D scanner among the 3 observers^1^

Measurement	Interobserver reliability					Bland–Altman agreement							
	Observer pairs	ICC	95% CI	*P*-value	Reliability	Bias	95% CI	ULOA	95% CI	LLOA	95% CI	*P*-value	% cows in LOA
Vertical	A–3D	0.96	0.94–0.97	<0.01	Excellent	−0.11	−0.56 to 0.35	4.62	3.84 to 5.4	−4.83	−5.61 to −4.05	0.65	96
	B–3D	0.90	0.85–0.93	<0.01	Good	0.29	−0.36 to 0.94	7.08	5.95 to 8.2	−6.49	−7.61 to −5.37	0.38	96
	C–3D	0.91	0.87–0.94	<0.01	Excellent	−0.65	−1.34 to 0.04	6.51	5.32 to 7.69	−7.81	−9.0 to −6.63	0.06	96
Right quarter	A–3D	0.92	0.89–0.95	<0.01	Excellent	1.04	0.63 to 1.45	5.3	4.6 to 6	−3.22	−3.93 to −2.52	<0.01	96
	B–3D	0.92	0.89–0.95	<0.01	Excellent	−0.56	−0.96 to −0.16	3.61	2.92 to 4.29	−4.73	−5.42 to −4.04	<0.01	96
	C–3D	0.89	0.84–0.92	<0.01	Good	0.75	0.24 to 1.25	5.92	5.06 to 6.77	−4.43	−5.28 to −3.57	<0.01	94
Left quarter	A–3D	0.88	0.82–0.92	<0.01	Good	0.75	0.3 to 1.2	5.4	4.63 to 6.17	−3.9	−4.7 to −3.13	<0.01	94
	B–3D	0.88	0.83–0.92	<0.01	Good	−0.79	−1.22 to −0.36	3.64	2.91 to 4.37	−5.22	−5.95 to −4.49	<0.01	93
	C–3D	0.87	0.81–0.91	<0.01	Good	0.42	−0.06 to 0.89	5.31	4.5 to 6.12	−4.48	−5.29 to −3.67	0.08	96

1 ICC = intraclass correlation coefficient; ULOA = upper limit of agreement; LLOA = lower limit of agreement; LOA = limit of agreement.

**Figure 1:**
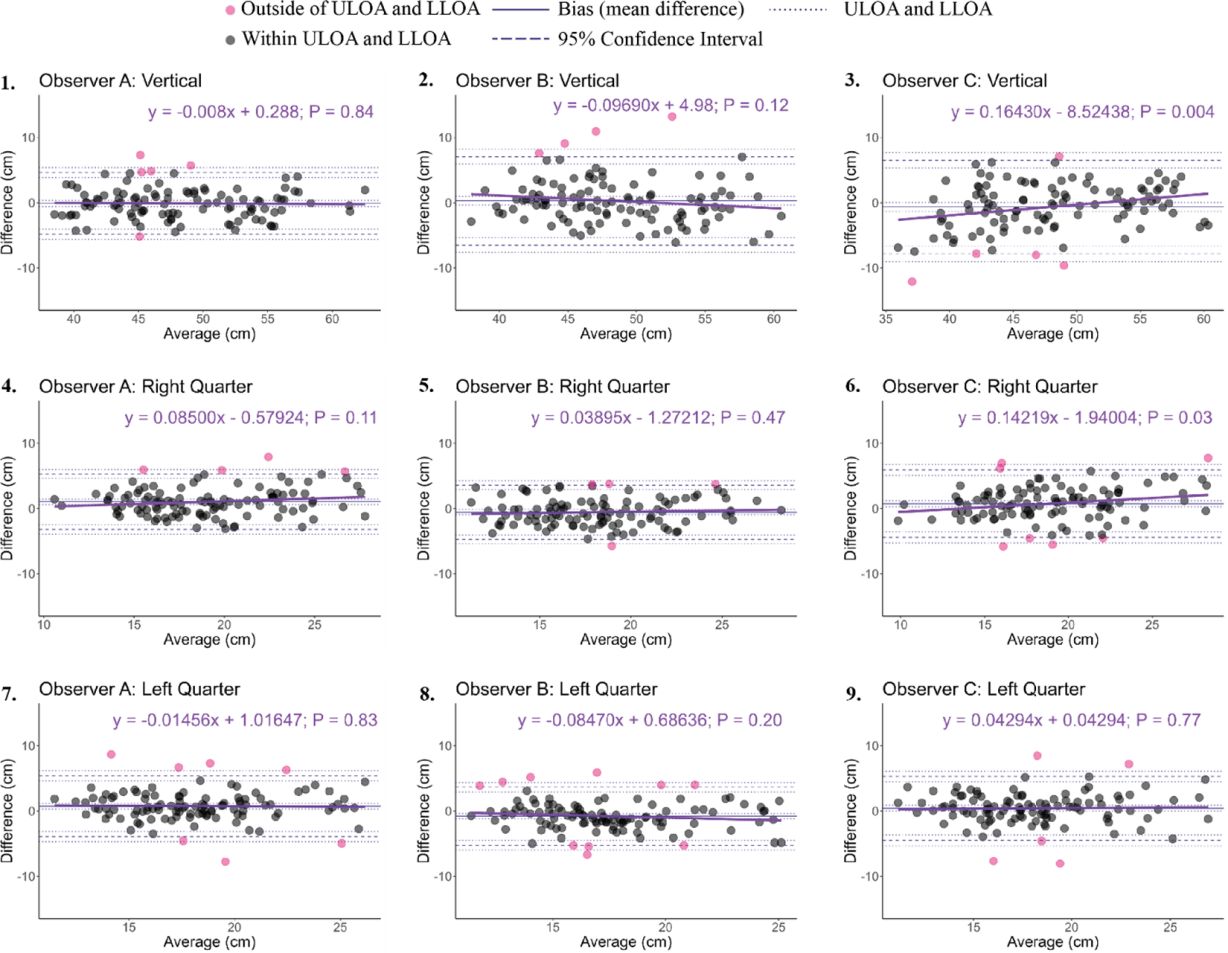
Bland–Altman plots between traditional and 3D measurements and linear regression models using Bland–Altman plot point data to investigate proportional bias. The y-axis is the difference between traditional and 3D measurements for each observer for the 3 measurements. The x-axis is the average of the traditional and 3D measurements. Linear regression models were created using the difference between traditional and 3D measurements as the dependent variable and the average of traditional and 3D measurements as the independent variable. ULOA = upper limit of agreement; LLOA = lower limit of agreement.

A study previously conducted by our group investigated the effects of pegbovigrastim dosed at dry-off on mammary gland health and mammary gland involution (de Campos et al., 2023). Mammary gland engorgement after dry-off was assessed with traditionally collected measurements and 3D scans using a similar method to this study. In the previous study, there was no difference in the width of the quarters between measurements collected traditionally and those measured by 3D scanner 2, 7, or 14 d after dry-off (de Campos et al., 2020). Due to the small sample size and study design, assessing reliability between measurement techniques was not possible.

Infrared thermography cameras that determine udder skin temperature are commonly used as imaging technology for mastitis research (Korelidou et al., 2024). Due to the inflammation that occurs as a result of bacterial infection in the mammary gland, increased udder skin temperature of the affected quarter can be useful in identifying infected cows, but results are influenced by ambient temperature (Wollowski et al., 2019). We will assess the potential of 3D scans for mastitis diagnosis in future studies.

The type of 3D scanner can affect how well images are collected in different environments (Bartol et al., 2021). The scanner used in our study is a structured light scanner that is based on triangulation and uses a speckle pattern of infrared light (Redaelli et al., 2018). The 3D scanner used most commonly in previous studies (Wang et al., 2023) is the Microsoft Kinect 1 that uses near-infrared camera similar to the Structure Sensor, but the Structure Sensor has 4 times the resolution (Redaelli et al., 2018, 2021). The increased resolution of the Structure Sensor results in decreased deviations in measurement compared with other sensors (Redaelli et al., 2018). However, the Kinect 1 could be mounted in fixed positions for udder morphometric data collection, which may be more practical on commercial farms compared with handheld devices that require closer proximity such as the Structure Sensor (Martins et al., 2020; Shorten, 2021). The objective of the current study was to assess the reliability of using the Structure Sensor to collect udder measurements in a research setting. However, these methods could potentially be adjusted for use on commercial dairies in settings where scans could occur in close proximity to stationary cows such as in robotic milking units.

The use of 3D scanners has become an accessible and accurate means of obtaining human body shape and size data and they are being increasingly used on dairy farms (Haleem and Javaid, 2019). In addition to convenience and the ability to automate data collection and processing, 3D scanners may reduce error inherent in collecting traditional measurements. There are 3 sources of error involved in collecting anatomical measurements on cattle: (1) identification of the correct reference points to begin and end a measurement, (2) changes in an animal's stance that can result in anatomical distortion, and (3) general error in measurement that can occur due to variation in tautness of a tape measure (Fisher, 1975). To avoid these sources of error, we marked the cow udders for uniform reference points, repositioned cows to stand squarely, and addressed tape tautness in our training. The observers collecting traditional measurements in this study had good or excellent interobserver reliability among each other and between the individuals and the 3D scanner. However, that might not be the case if anatomical landmarks were not predetermined by one individual. A focus on intra- and interrater agreement on anatomical landmarks should be considered in future studies. One potential source of error not investigated in the study is the potential error in collecting the measurements using the 3-matic software. An individual uses anatomical points of interest to select the beginning and end points of measurements on the 3D images. There is potential for variation in the identification of reference points and from anatomical distortion due to variation in an animal's stance during the scan. These potential sources of variation in measurements taken on 3D images are similar to those that can occur with traditionally collected measurements. Future research should include an assessment of the reliability among different observers collecting measurements using images collected with a 3D scanner. Another limitation of this study was that we did not perform a complete validation study using the 3D scanner, which increases the risk for biased results. However, we see this initial study as a promising step toward future studies toward validation by following the Consensus-based Standards for the Selection of Health Status Measurement Instruments guidelines (Mokkink et al., 2010). Finally, there is no gold standard for udder measurement collection. Without a gold standard in our study, we were unable to assess the accuracy of the traditionally collected or 3D scan measurements.

In this study, we observed good to excellent reliability and adequate agreement with minimal bias among all the observers and between observers and the 3D scanner. These results indicate that a 3D scanner can capture reliable measurements of the rear udder and may be useful in future udder health studies.
